# Silence of MACC1 expression by RNA interference inhibits proliferation, invasion and metastasis, and promotes apoptosis in U251 human malignant glioma cells

**DOI:** 10.3892/mmr.2015.3886

**Published:** 2015-06-03

**Authors:** LONGFENG SUN, GANG LI, BING DAI, WEI TAN, HONGWEN ZHAO, XIAOFEI LI, AIPING WANG

**Affiliations:** 1 Department of Respiratory Medicine, The First Affiliated Hospital of China Medical University, Shenyang, Liaoning 110001, P.R. China; 2 Department of Urology, Liaoning Cancer Hospital and Institute, Shenyang, Liaoning 110042, P.R. China; 3 Departments of Emergency Medicine, The First Affiliated Hospital of China Medical University, Shenyang, Liaoning 110001, P.R. China; 4 Nursing, The First Affiliated Hospital of China Medical University, Shenyang, Liaoning 110001, P.R. China

**Keywords:** U251 cells, metastasis-associated in colon cancer 1, proliferation, apoptosis, invasion, metastasis

## Abstract

The overexpression of metastasis-associated in colon cancer 1 (MACC1) has been demonstrated not only in colon cancer, but also in various other types of cancer. Gliomas are the most common type of intracranial tumors, and recent studies have reported MACC1 to be involved in human glioma progression. The present study aimed to investigate the effects of MACC1 expression silencing in glioma cells using RNA interference, in order to determine the underlying biological mechanisms of glioma progression, including proliferation, apoptosis, invasion and metastasis. The expression levels of MACC1 were determined in various types of U251 glioma cells using western blot analyses. MACC1-specific short hairpin RNA (shRNA) was used to silence the expression of MACC1 in the U251 cells. The results obtained following MACC1 silencing demonstrated a significant inhibition of cell proliferation, invasion and migration, as well as a marked enhancement of apoptosis. MACC1 shRNA-induced inhibition of cell proliferation was observed by colony forming and MTT assays, and cell apoptosis was measured using flow cytometry and Hoechst staining. In addition, inhibition of cell invasion and migration was assessed using wound healing and transwell assays. Western blotting and fluorescence-activated cell sorting (FACS) revealed a G_0_/G_1_ phase cell cycle arrest regulated by cyclins D1 and E; cell apoptosis regulated by caspase-3; and cell invasion and migration regulated by matrix metalloproteinases 2 and 9, respectively. The present study demonstrated that the expression levels of MACC1 were significantly correlated with the biological processes underlying glioma cell proliferation, invasion and metastasis. Therefore, MACC1 may serve as a promising novel therapeutic target in human glioma. Notably, the inhibition of MACC1 expression by shRNA may prove to be an effective genetic therapeutic strategy for glioma treatment.

## Introduction

Gliomas are the most common type of intracranial tumor, with malignant gliomas considered to be among the most severe, not only due to their poor prognosis, but also due to their direct repercussions on patient cognitive function and quality of life (1,2). In the United States, the annual incidence of malignant glioma is 6.04 per 100,000 people (3), and in Europe, the annual incidence is 3–5 per 100,000 people (4). Despite modern diagnostics and marked advances in surgical techniques, as well as systemic chemotherapy treatments, the prognosis for patients with malignant glioma remains poor, with a median survival rate of <15 months (5). Therefore, the study of the mechanisms underlying tumorigenesis and glioma progression is paramount to the development of novel chemotherapeutics, and for the identification of novel therapeutic targets for malignant glioma treatment.

The metastasis-associated in colon cancer 1 (MACC1) gene was initially identified in a genome-wide search for differentially expressed genes in human colon cancer primary tissue samples, metastatic tissue samples, and normal tissue samples (6). Previous studies have suggested that MACC1 is expressed not only in colon cancer, but also in various other human cancers, including lung cancer (7,8), hepatocellular carcinoma (9), ovarian carcinoma (10) and gastric carcinoma (11). In addition, further studies have demonstrated that MACC1 is associated with the proliferation, invasion, metastasis, and survival of tumor cells in these cancer types (12). Furthermore, overexpression of MACC1 serves as an independent indicator of patient prognosis, by predicting the rates of recurrence and disease-free survival (13). Yang *et al* (14) previously reported that MACC1 protein was overexpressed in glioma (14). In addition, Hagemann *et al* (15) hypothesized that MACC1 may be involved in the progression of human malignant glioma, as its overexpression is associated with poor patient prognosis. However, the mechanisms underlying the role of MACC1 in glioma remain unclear, and the impact of MACC1 on proliferation, invasion, metastasis and survival has yet to be fully understood.

The present study aimed to investigate the effects of MACC1 on cell inhibition, proliferation, apoptosis, invasion, and metastasis in human U251 glioma cells, following transfection with MACC1-specific short hairpin RNA (shRNA) expression plasmids.

## Materials and methods

### Cell lines

The U373, U251, A172, U87-MG and SHG4 human malignant glioma cell lines used in the present study were purchased from the American Type Culture Collection (Manassas, VA, USA). All of the cell lines were cultured in Dulbecco's modified Eagle's medium (DMEM; Gibco Life Technologies, Carlsbad, CA, USA) supplemented with 100 units/ml penicillin, 100 *µ*g/ml streptomycin, and 10% (v/v) heat-inactivated fetal bovine serum (FBS; GE Healthcare Life Sciences, Logan, UT, USA) at 37°C. The medium was renewed every 2–3 days.

### Cell transfection

The confluent cells (80–90% confluent) were harvested for transfection. Lipofectamine^®^ 2000 Transfection reagent, and nonsense and MACC1-specific shRNA (InvitrogenLife Technologies, Carlsbad, CA, USA) were diluted in Opti-MEM^®^ medium (Life Technologies, Grand Island, NY, USA). The shRNA sequences were as follows: MACC1-specific shRNA, atggcttggttaagtcaac; and nonsense shRNA, ttctccgaacgtgtcacgt. The shRNA was added to the Lipofectamine^®^ 2000 Transfection Reagent at a 1:1 ratio, prior to being incubated for 5 min at room temperature. The shRNA-lipid complex was then added to the cells, and further incubated for 1 day at 37°C. The transfected cells were cultured in DMEM supplemented with Geneticin^®^ (Gibco Life Technologies) for 1–2 weeks. Transfection efficiency was assessed by fluorescence microscopy (DP73; Olympus Corporation, Tokyo, Japan).

### Reverse transcription-quantitative polymerase chain reaction (RT-qPCR)

RNA was extracted from the experimental cells using the RNA Simple Total RNA kit (Tiangen Biotech Co., Ltd., Beijing, China), according to the manufacturer's instructions. Briefly, in order to synthesize the cDNA, total RNA (1 *µg*) was reverse transcribed in 20 *µ*l 2X Power Taq PCR MasterMix (BioTeke Corporation, Beijing, China) according to the manufacturer's instructions. The RT products were subsequently amplified using RT-qPCR. The primers used for RT-qPCR were as follows: MACC1 sense, 5′-CACAACTTGCGGAGGTCAC-3′; antisense, 5′-TTCCAACAACGGGCTCACAG-3′; and β-actin sense, 5′-CTTAGTTGCGTTACACCCTTTCTTG-3′; antisense, 5′-CTGTCACCTTCACCGTTCCAG TTT-3′ (Sangon Biotech Co., Ltd., Shanghai, China). The RT was performed using the Exicycler 96 Research system (Bioneer Corporation, Daejeon, Korea), and the reaction conditions for the RT were as follows: 25°C for 10 min, 42°C for 50 min, and 95°C for 5 min. The results of the PCR were analyzed using the 2^−ΔΔCt^ method (16).

The PCR amplification conditions were as follows: 95°C for 10 min, followed by 40 cycles of 95°C for 10 sec, 60°C for 20 sec and 72°C for 30 sec, and 4°C for 5 min. The results of the PCR were verified by varying the number of PCR cycles for each cDNA, and for each set of primers. The RT-qPCR was performed in triplicate.

### Western blot analysis

The cells were harvested and lysed using radioimmunoprecipitation buffer (Beyotime Institute of Biotechnology, Haimen, China) and phenylmethylsulfonyl fluoride (Beyotime Institute of Biotechnology) for 30 min on ice. The protein extracts were subsequently centrifuged at 24,148 × g for 10 min at 4°C, and the protein concentration was determined using a bicinchoninic acid protein assay kit (Beyotime Institute of Biotechnology). The protein extracts (40 *µ*g) were subsequently separated by 8%–12% SDS-PAGE prior to being transferred onto polyvinylidene fluoride membranes (EMD Millipore, Billerica, MA, USA). The membranes were then stained with 0.2% Ponceau S Red (Beyotime Institute of Biotechnology) to ensure equal loading of the proteins. The membranes were then blocked with 5% nonfat milk, and were incubated with antibodies targeting MACC1 (1:100; cat. no. bs-4293R; BIOSS, Beijing, China), and cyclin D1 (1:1,000; cat. no. WL0205), cyclin E (1:1,000; cat. no. WL0055), cyclin B (1:1,000; cat. no. WL0023), cleaved caspase-3 (1:200; cat. no. WL0146), B cell lymphoma 2 (Bcl-2; 1:500; cat. no. WL0104), Bcl-2-associated X protein (Bax; 1:500; cat. no. WL0101), matrix metalloproteinase 2 (MMP-2; 1:500; cat. no. WL0657), MMP-9 (1:500; cat. no. WL0884) and β-actin (1:1,000; cat. no. WL0001a; all Wanleibio Ltd., Shenyang, China) overnight at 4°C. The membranes were subsequently incubated with horseradish peroxidase-conjugated goat anti-rabbit immunoglobulin G secondary antibody at 1:5,000 dilution, at room temperature for 1 h (cat. no. A0208; Beyotime Institute of Biotechnology). β-actin was used as the internal positive control. The immunocomplexes were visualized using enhanced chemiluminescence western blotting detection reagents (7seabiotech, Shanghai, China). The relative amounts of transferred protein were quantified by scanning the autoradiographic films using UN-SCAN-IT Gel Analysis software (Silk Scientific Inc., Orem, UT, USA) prior to being normalized to the corresponding β-actin level. The quantitative analysis of the western blot was carried out using the Gel-Pro Analyzer software (Media Cybernetics, Inc., Rockville, MD, USA).

### Apoptosis and cell cycle assays

In order to conduct the apoptosis assay, the comparative levels of apoptotic cells were determined by fluorescence-activated cell sorting (FACS), following staining with propidium iodide (PI) and Annexin V. Briefly, the cells were harvested and washed twice in cold phosphate buffered saline (PBS; Hyclone, Logan, UT, USA) prior to being stained with PI, and subsequently detected using the Annexin V-Fluorescein Isothiocyanate Apoptosis Detection kit (Nanjing KeyGen Biotech Co., Ltd, Nanjing, China), according to the manufacturer's instructions. Flow cytometry was performed using the BD FACSCalibur (BD Biosciences, San Jose, CA, USA), and the data were analyzed using FCS Express software version 3 (BD Biosciences).

For the cell cycle assay, the cells were fixed with ice-cold 70% ethanol at a density of 1×10^5^ cells/ml, and treated with 200 *µ*g/ml ribonuclease (Beyotime Institute of Biotechnology) for 30 min at 37°C. PI (Beyotime Institute of Biotechnology) was subsequently added to the solution in order to produce a final concentration of 50 *µ*g/ml. The DNA content was then quantitated by flow cytometry (FACSCalibur; BD Biosciences) with an excitation wavelength of 488 nm, and an emission wavelength of 625 nm. The data were analyzed using CellQuest (BD Biosciences) software on 10,000 events.

### Colony forming assay

The cells were seeded onto 35 mm cell culture dishes (200/plate; Corning Life Sciences, Manassas, VA, USA) and cultured in DMEM supplemented with 10% FBS. The medium was changed every 3 days, and following a 14 day incubation the cells were fixed with 4% paraformaldehyde for 5–8 min prior to being stained with Wright-Giemsa (Nanjing Jiancheng Bioengineering Institute, Nanjing, China). The stained cells were observed, and images were captured using a stereomicroscope (AE31; Motic Microscopes, Xiamen, China). The experiment was repeated three times, and aggregates with >50 cells were scored as a colony. Colony forming efficiency was determined using the following formula: Colony forming efficiency = (colony number in sh(MACC1)/cell population) x 100%.

### MTT colorimetric assay

The cells were seeded onto 96-well plates at a density of 2×10^3^ cells/well and incubated in DMEM supplemented with 10% serum for 24, 48, 72, and 96 h, prior to being analyzed for cell growth. Five duplicate wells were set up for each group. At each time point, 0.2 mg/ml MTT (Sigma-Aldrich, St. Louis, MO, USA) was added to each well and incubated for 4 h, following which 200 *µ*l dimethyl sulfoxide (Sigma-Aldrich) was added to each well. Following the complete solubilization of the dye, the absorbance levels of the wells were measured using a microplate spectrophotometer (ELx-800; BioTek Instruments, Inc., Winooski, VT, USA) at 490 nm.

### Hoechst staining assay

The cells were seeded onto 12-well plates at a density of 1×10^5^ cells/well and incubated in DMEM containing 10% FBS for ~24 h. Once the cells grew to occupy 80% of the well, they were fixed with a stain-fixative (Beyotime Institute of Biotechnology) for 20 min. The fixed cells were subsequently washed twice in cold PBS for 3 min prior to being stained with Hoechst (Beyotime Institute of Biotechnology) for a further 5 min. Morphological changes of the cellular cytoskeleton were observed using a fluorescence microscope (Olympus Corporation).

### Transwell assay

Transwell chambers were used to measure cell migration. A total of 200 *µ*l cells in serum-free DMEM were seeded into each upper chamber (Corning Life Sciences, Manassas, VA, USA) of the transwell at a concentration of 2×10^4^ cells/ml, whereas 800 *µ*l cells were seeded into each lower chamber in DMEM supplemented with 15% FBS, which acted as a chemoattractant. Three duplicate wells were set up for each group. The cells were subsequently removed from the upper surface of the filter following a 24 h culture. The cells that penetrated through the filter were fixed with 4% paraformaldehyde for 20 min, prior to being stained with 0.5% crystal violet (Amresco LLC, Solon, OH, USA). Five non-overlapping random visual fields were selected in order to count the cells on the lower membrane under a high powered lens (x200) (AE31; Motic Microscopes).

### Wound healing assay

The cells were seeded onto 6-well plates and cultured until confluent (80–90%) in DMEM supplemented with 10% FBS. The medium was discarded and a straight scratch was subsequently placed across the culture, using a 200 *µ*l pipette tip, in order to simulate a wound. The plates were then washed twice with serum-free DMEM. The cells were observed and images were captured using a microscope (AE31; Motic Microscopes), in order to ensure enough cells were present at the leading edge of the wound. The cells were subsequently cultured in serum-free DMEM for a further 12–24 h prior to image capture. The migration rate of the cells was calculated by measuring the distance travelled by the cells towards the center of the wound at 12 and 24 h.

### Gelatin zymography assay

Gelatin zymography was performed in order to determine the activity of MMP-2 and MMP-9. Briefly, the protein samples (40 *µ*g) were separated by 10% SDS-PAGE containing 1 mg/ml gelatin (Sigma-Aldrich) at 4°C. Following electrophoresis, the gel was incubated with 2.5% Triton X-10 0 (Amresco LLC) in distilled water with gentle agitation for 40 min at room temperature. Subsequently, the gel was further incubated in developing buffer (50 mM Tris-HCl, 0.2 M NaCl, 5 mM CaCl_2_, 1 *µ*M ZnCl_2_ and 0.02% Brij35) overnight with gentle agitation. The gel was then stained with Coomassie blue R-250 (Amresco LLC) for 30 min. Following incubation with the staining solution, the gel was washed with a destaining solution (2.5% Triton X-100, 50 mmol/l Tris-HCl, 5 mmol/l CaCl_2_, 1 *µ*mol/l ZnCl_2_; pH 7.6) until the bands became visible against the blue background. The incubation time was optimized depending on the enzyme activity. The quantitative analysis of the gelatin zymography was carried out using the Gel Documentation & Analysis system (Beijing Liuyi Instrument Factory, Beijing, China).

### Statistical analysis

The data from a minimum of three experiments were presented as the mean ± standard deviation. Statistical comparisons were subsequently made using the Bonferroni multiple comparison test, or with a one-way analysis of variance. SPSS version 20.0 (IBM SPSS, Armonk, NY, USA) was used to conduct statistical analyses. P<0.05 was considered to indicate a statistically significant difference.

## Results

### Screening of MACC1 expression levels in various glioma cell lines, and the establishment of glioma cell lines with silenced MACC1 expression

**Figure 1:**
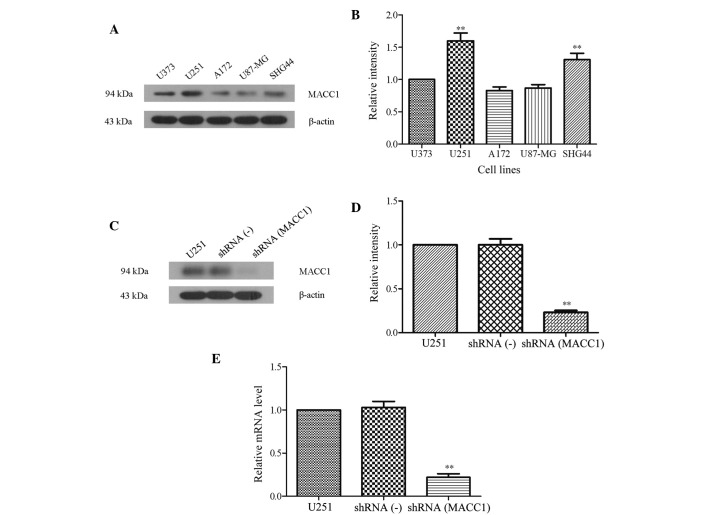
Screening of the metastasis-associated in colon cancer 1 (MACC1) expression levels in various glioma cell lines, and establishment of glioma cell lines containing silenced MACC1. (A) Protein expression levels of MACC1 were highest in the U251 cells, as compared with the other glioma cell lines. The expression levels of MACC1 in the U373, U251, A172, U87-MG and SHG44 cells were determined using western blot analysis during the cellular growth phase, and (B) the quantitative analysis of gray intensity was determined. ^**^P<0.01, vs. A172 group. (C) MACC1 silencing in the U251 cells was established using RNA interference (RNAi). The relative protein expression levels of MACC1 in the U251, small hairpin (sh)RNA(-), and shRNA(MACC1) cells were determined by western blot analysis, and (D) the quantitative analysis of gray intensity was calculated for each cell line. No marked differences were observed between the U251 and shRNA(-) cells. ^**^P<0.01, vs. shRNA(-) group. (E) Relative mRNA expression levels of MACC1 were determined by reverse transcription-quantitative polymerase chain reaction in the U251, shRNA(-), and shRNA(MACC1) cells. ^**^P<0.01, vs. shRNA(-) group. Data are presented as the mean ± standard deviation. U251, U251 cells without RNAi; shRNA(-), U251 cells transfected with negative control shRNA; shRNA(MACC1), U251 cells transfected with silenced MACC1 shRNA.

The expression levels of MACC1 were detected in various glioma cell lines by western blotting. As demonstrated in Fig. 1A, the protein expression levels of MACC1 were highest in U251 glioma cells. Quantitative analysis of the gray intensity revealed that the relative intensity of MACC1 in the U251 cells was 1.60±0.21, almost twice the relative intensity of MACC1 in A172 cells (0.83±0.10). The relative intensity of MACC1 expression in the glioma cell lines was as follows: U251 (1.60±0.21), SHG44 (1.31±0.17), U373 (1.00±0.00), U87-MG (0.87±0.09), and A172 (0.83±0.10) (Fig. 1B; P<0.01). These results suggest that the expression levels of MACC1 were markedly higher in U251 cells, as compared with other glioma cell lines such as A172 and U87-MG (P<0.01). Therefore, the U251 cell line was selected to investigate the effects of MACC1 silencing on the proliferation and metastasis of malignant glioma cells.

The interference fragments of the MACC1 gene group and the nonsense oligodeoxynucleotide group were designed and synthesized, prior to being ligated into pGCsi-H1 plasmids. The pGCsi-H1 plasmid constructs were subsequently transfected into the U251 cell lines, in order to generate shRNA(-), and shRNA(MACC1) U251 cells. To examine stable transfection efficiency, a western blot analysis was used to determine the expression levels of MACC1 in the U251, shRNA(-) U251, and shRNA(MACC1) U251 cells. As shown in Fig. 1C, there was little MACC1 expression in the shRNA(MACC1) U251 cells. Although there was no marked difference between the expression levels of MACC1 in the shRNA(-) U251 and the U251 cells, the quantitative analysis of gray intensity revealed that MACC1 expression levels were 4-fold lower in the shRNA (0.23±0.04) cells, as compared with the U251 (1.00±0.00) and shRNA(-) (1.00±0.12) cells (Fig. 1D; P<0.01). In addition, RT-qPCR was used to determine the mRNA expression levels of MACC1 in the three cell lines. The results of the RT-qPCR demonstrated a similar trend to those of the western blot analysis. The mRNA expression levels of MACC1 were also 4-fold lower in the shRNA cells (0.22±0.07), as compared with the U251 (1.0±0.00) and shRNA(-) (1.03±0.12) cells (Fig. 1E, P<0.01). These data suggest that successful subclones of the MACC1 silenced U251 cells [shRNA (MACC1)], and the negative shRNA(-) cells were generated in the present study.

### Silencing of MACC1 expression inhibits cell proliferation and induces cell cycle arrest in U251 cells

**Figure 2:**
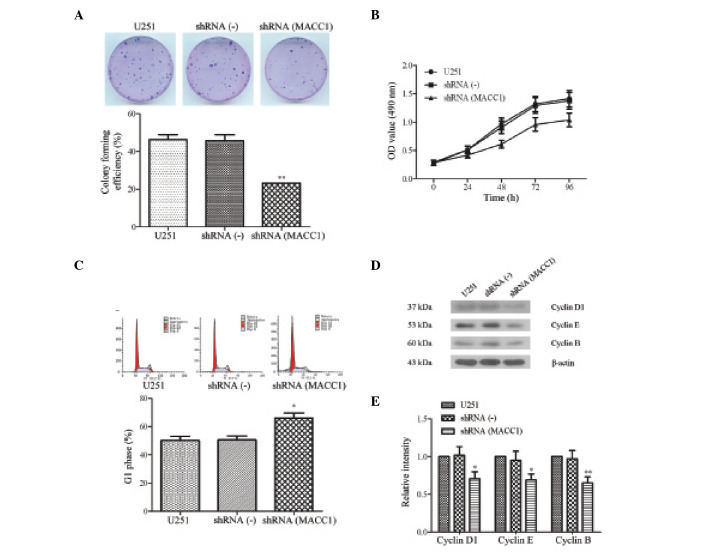
Silencing of metastasis associated in colon cancer 1 (MACC1) expression inhibited cell proliferation, and induced cell cycle arrest in U251 human glioma cells. (A) Colony formation rate was decreased by MACC1 silencing in small hairpin (sh)RNA(MACC1) U251 cells. (A, upper panel) The number of colonies in the shRNA(MACC1) cells was much lower, as compared with the number of colonies in the U251 and shRNA(-) cells. (A, lower panel) Colony forming efficiency was calculated. ^**^P<0.01, vs. shRNA(-) group. (B) Suppression of cell proliferation by MACC1 shRNA in the U251 cells was measured using an MTT assay. The proliferation of U251 cells was significantly inhibited in a time-dependent manner following MACC1 silencing. ^**^P<0.01, vs. shRNA(-) group. (C) Cell cycle arrest in the U251 cells was induced by MACC1 shRNA. Changes in the U251 cell cycle induced by MACC1 shRNA were examined using fluorescence-activated cell sorting. (C, upper panel) There was an increase in shRNA(MACC1) cells at G_0_/G_1_ phase; (C, lower panel) The ratio of U251, shRNA(-) and shRNA(MACC1) cells in G_0_/G_1_ phase was calculated. ^*^P<0.05, vs. shRNA(-) group. (D) Expression levels of cell cycle arrest-associated proteins cyclin D1, cyclin E and cyclin B were examined and compared by western blot analysis, and (E) the quantitative analysis of gray intensity was calculated. ^*^P<0.05, ^**^P<0.01, vs. shRNA(-) group. Data are presented as the mean ± standard deviation. U251, U251 cells without RNA interference; shRNA(-), U251 cells transfected with negative control MACC1 shRNA; shRNA(MACC1), U251 cells transfected with silenced MACC1 shRNA.

In order to determine the effects of MACC1 silencing on U251 cell proliferation and cycle, a colony forming assay was used to determine the proliferation of shRNA(MACC1) cells, as compared with the proliferation of shRNA(-) and U251 cells. As shown in Fig. 2A, following a 14 day incubation the number of shRNA(MACC1) cell aggregates was much lower, as compared with the shRNA(-) and U251 cells (Fig. 2A). In addition, a colony forming efficiency assay revealed that the efficiency of the shRNA(MACC1) cells was half that of the shRNA(-) or U251 cells (23.20±4.37, vs. 45.80±5.37% or 23.20±4.37, vs. 46.20±4.70%; P<0.01; Fig. 2A, lower panel).

Furthermore, an MTT assay demonstrated that the proliferation of the shRNA(MACC1) cells was inhibited in a time-dependent manner, as compared with the shRNA(-) and the U251 cells (Fig. 2B). These results suggest that the knockdown of MACC1 expression by shRNA may inhibit the growth of U251 cells.

To investigate the signaling pathway of growth inhibition induced by MACC1 silencing, the cell cycle was examined using FACS analysis following PI staining in the shRNA(MACC1), shRNA(-) and U251 cells. The results of the experiment indicated that the G_0_/G_1_ phase ratio increased from 50.18±4.97 to 66.07±6.07% (P<0.05) in the U251 cells following transfection with MACC1 shRNA (Fig. 2C). These results suggest that MACC1 shRNA may inhibit cell proliferation by interfering with cell mitosis and inducing cell cycle arrest.

To further investigate the mechanism underlying MACC1 shRNA-induced cell cycle arrest, cell cycle-associated proteins: Cyclin D1, cyclin E and cyclin B, were analyzed using western blotting. The results demonstrated that the protein expression levels of cyclin D1, cyclin E and cyclin B were downregulated in the shRNA(MACC1) cells as compared with the shRNA(-)or U251 cells (Fig. 2D). No marked difference existed between the shRNA(-) and the U251 cells. The quantitative value of gray intensity of cyclin D1, cyclin E and cyclin B, between the shRNA(MACC1) and the shRNA(-) cells was determined to be 0.71±0.09, vs. 1.02±0.11, 0.69±0.08, vs. 0.95±0.12, and 0.65±0.08, vs. 0.97±0.11, respectively (P<0.05 and P<0.01, Fig. 2E). These results indicate that MACC1 shRNA was able to inhibit cell proliferation by inducing cell cycle arrest, and by downregulating cyclin D1, cyclin E and cyclin B expression.

### MACC1 silencing induces apoptosis in U251 cells

**Figure 3:**
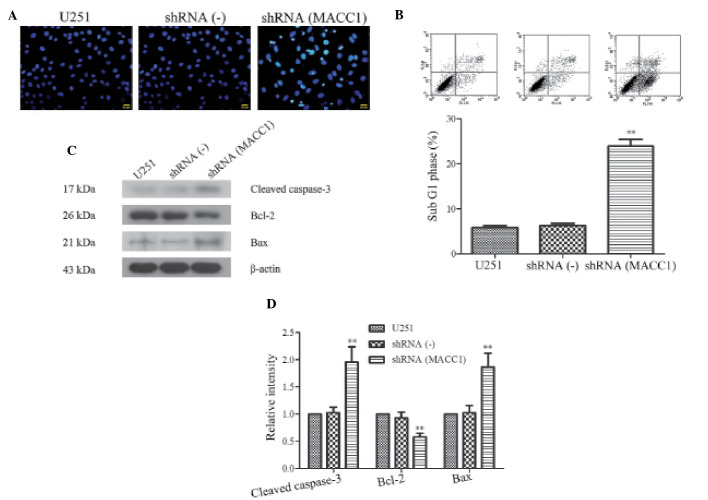
Silencing of metastasis associated in colon cancer 1 (MACC1) expression induced apoptosis in U251 human glioma cells. (A) Apoptosis was induced by transfection with MACC1 small hairpin (sh)RNA. The apoptotic shRNA(MACC1) cells were observed using Hoechst staining following 24 h incubation (scale bar=20 *µ*m). (B) The number of apoptotic cells was determined by fluorescence activated cell sorting following staining with Annexin V-fluorescein isothiocyanate. (B, upper panel) Apoptotic cells were more numerous in the shRNA(MACC1) cells, as compared with the U251 and shRNA(-) cells, and (B, lower panel) the apoptotic index was calculated. ^**^P<0.01, vs. shRNA(-) group. (C) The expression levels of apoptosis-associated proteins. The expression levels of cleaved caspase-3, B cell lymphoma 2 (Bcl-2), and Bcl-2 associated X protein (Bax) in the U251, shRNA(-) and shRNA(MACC1) cells were examined using western blot analysis; (D) the quantitative analysis of gray intensity was calculated. ^**^P<0.01, vs. shRNA (-) group. Data are presented as the mean ± standard deviation. U251, U251 cells without RNA inteference; shRNA(-), U251 cells transfected with negative control MACC1 shRNA; shRNA(MACC1), U251 cells transfected with silenced MACC1 shRNA.

The present study evaluated the correlation between MACC1 silencing and apoptosis in U251 cells. The number of apoptotic cells was determined by morphologic observation following Hoechst staining, and the morphological changes were observed by fluorescence microscopy. The results indicated that apoptosis occurred in the shRNA(MACC1) cells following a 24 h incubation (Fig. 3A). Subsequently, the apoptotic cells were quantified by FACS analysis, using the Annexin V-FITC Apoptosis Detection kit (Fig. 3B). The apoptotic index of the shRNA(MACC1) cells (23.90±2.64%) was markedly higher as compared with the shRNA(-) cells (6.31±0.88%) and the U251 (5.84±0.81%) cells (P<0.01, Fig. 3B). These results indicate that MACC1 shRNA was able to induce apoptosis in U251 cells.

In order to determine the underlying mechanisms of MACC1 shRNA-induced apoptosis, the levels of apoptosis-associated proteins were detected in the U251, shRNA(-) and shRNA(MACC1) cells by western blot analysis. The detection of high expression levels of cleaved caspase-3 suggested the existence of a caspase-mediated pathway that may lead to apoptosis. Apoptotic pathway activation is regulated by the balance between pro-survival proteins, such as Bcl-2, and pro-apoptotic proteins, such as Bax. Therefore, the protein expression levels of Bcl-2 and Bax were investigated in the three cell lines. The results demonstrated that the levels of the anti-apoptotic protein Bcl-2 were downregulated in the shRNA(MACC1) cells, whereas the levels of pro-apoptotic protein Bax were upregulated (Fig. 3C). There were no marked differences between the shRNA(-) and U251 cells. The quantitative value of gray intensity of cleaved caspase-3, Bcl-2, and Bax, between the shRNA(MACC1) and shRNA(-) cells was determined as 1.96±0.28, vs. 1.03±0.10, 0.58±0.07, vs. 0.93±0.11, and 1.87±0.25, vs. 1.03±0.13, respectively (P<0.01; Fig. 3D). These results suggest that the upregulation of Bax and the downregulation of Bcl-2 are responsible for activation of the apoptotic pathway, following RNAi-mediated silencing of MACC1 expression.

### MACC1 shRNA suppresses the invasion and migration of U251 cells

**Figure 4:**
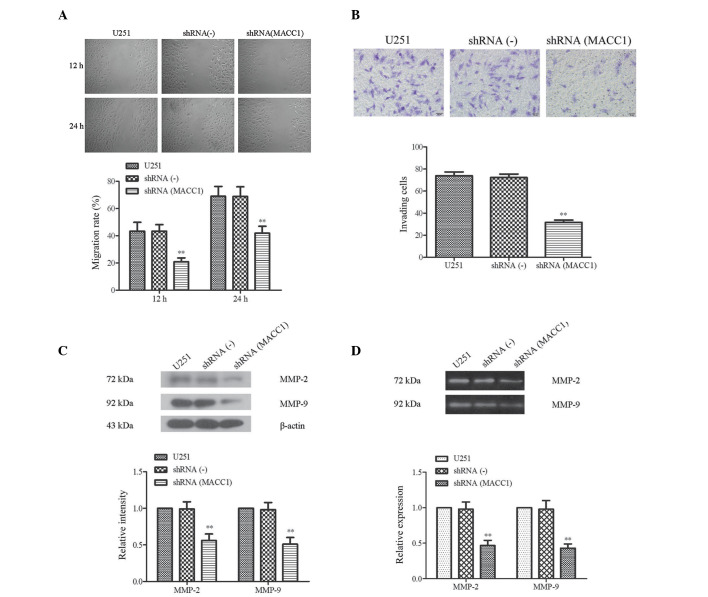
Suppression of cell invasion and migration induced by metastasis associated in colon cancer 1 (MACC1) short hairpin (sh)RNA. (A) Wound healing assay was conducted after silencing of MACC1 expression. (A, upper panel; scale bar=200 *µ*m) Migration rate was significantly inhibited in the shRNA(MACC1) cells, as determined by a wound healing assay, and (A, lower panel) the migration rate was quantified. ^**^P<0.01, vs. shRNA(-) group. (B, upper panel; scale bar=100 *µ*m) Transwell assay following silencing of MACC1 expression (B, lower panel) and the comparison of invasion capacity in the U251, shRNA(-) and shRNA(MACC1) cells. ^**^P<0.01, vs. shRNA(-) group. (C) Expression levels of invasion and migration-associated proteins. (C, upper panel) Protein expression levels of matrix metalloproteinase (MMP)-2 and MMP-9 in the U251, shRNA(-), and shRNA(MACC1) cells were tested and compared by western blot analysis; (C, lower panel) the quantitative analysis of gray intensity was calculated. ^**^P<0.01, vs. shRNA (-) group. (D) The activities of MMP-2 and MMP-9 were decreased by MACC1 shRNA. (D, upper panel) The activities of MMP-2 and MMP-9 were tested by gelatin zymography assay; and (D, lower panel) the relative activities of MMP-2 and MMP-9 were calculated. ^**^P<0.01, vs. shRNA(-) group. Data are presented as the mean ± standard deviation. U251, U251 cells without RNA interference; shRNA(-), U251 cells transfected with negative control MACC1 shRNA; shRNA(MACC1), U251 cells transfected with silenced MACC1 shRNA.

The results of the present study demonstrated that the suppression of cell invasion and migration was induced by MACC1 shRNA. In addition, a previous study reported that the expression of MACC1 is associated with the invasion and metastasis of various human cancers (17). Therefore, in order to determine whether MACC1 was necessary for cell migration, a wound healing assay was conducted to examine the migratory ability of the U251 cells following MACC1 silencing. The cells transfected with MACC1 shRNA exhibited decreased migratory ability following a 12–24 h incubation period (Fig. 4A). Notably, the distance of shRNA(MACC1) cell migration was markedly decreased. There were no marked differences between the shRNA(-) and U251 cells. The migration rate of the shRNA(MACC1) cells, vs. the shRNA(-) cells was determined to be 20.83±2.88%, vs. 43.37±7.44% for 12 h, and 41.93±5.12%, vs. 68.8±7.15% for 24 h, respectively (P<0.01; Fig. 4A).

A transwell assay was used to investigate the suppression of invasion by MACC1 shRNA. As compared with the control group, the shRNA(MACC1) cells demonstrated a suppressed capacity for invasion (Fig. 4B). Furthermore, the number of cells adhering to the lower membranes of the transwell chamber was markedly decreased in the shRNA(MACC1) group, which exhibited half the invasive capacity of the shRNA(-) and U251 groups (31.60±4.77, vs. 72.20±7.33 and 31.60±477, vs. 73.80±799, P<0.05) (Fig. 4B). These results suggest that MACC1 RNAi may suppress invasion and migration of malignant glioma cells.

Increased MMP-2 and MMP-9 activity levels are considered an important mechanism for the increased capacity of cancer cells to traverse the membrane, mimicking invasion and metastasis (18). Therefore, the present study analyzed the effects of MACC1 shRNA on the expression levels of MMP-2 and MMP-9 in the shRNA(MACC1), shRNA(-) and U251 cells using a western blot analysis. As shown in Fig. 4C, the expression levels of MMP-2 and MMP-9 were significantly decreased in the shRNA(MACC1) cells. A quantitative value of gray intensity analysis demonstrated that the gray intensity value of MMP-2/-9 in the shRNA(MACC1) cells was only half that of the shRNA(-) cells (P<0.01, Fig. 4C). In addition, the MMP-2 and MMP-9 activity levels were examined using a gelatin zymography assay. The results of the assay demonstrated that the enzymatic activities of MMP-2 and MMP-9 were inhibited in the shRNA(MACC1) group (Fig. 4D, upper panel). Further quantification analysis indicated that the MMP-2 and MMP-9 activity levels were reduced to 0.47±0.07, and 0.43±0.06 as compared with the shRNA(-) group where their activity levels were determined as 0.98±0.01, and 0.98±0.12, respectively (P<0.01; Fig. 4D, lower panel). These results indicate that MACC1 shRNA was able to markedly inhibit the activity of MMP-2 and MMP-9.

## Discussion

Numerous studies have previously shown that MACC1 is abnormally expressed in various human malignances, and that it acts as a metastatic pacemaker in colorectal, gastric and various other cancer cells (11,19,20). Chai *et al* (16) demonstrated that in HeLa cervical cancer cells, the downregulation of MACC1 resulted in the inhibition of cell proliferation, migration and invasion, and the enhancement of apoptosis. Furthermore, Meng *et al* (21) reported that MACC1 has an important role in the carcinogenesis of nasopharyngeal carcinoma cells through the activation of the Akt/β-catenin signaling pathway. However, the role of MACC1 in glioma cancer initiation and progression remains to be elucidated. In the present study, the expression levels of MACC1 were compared in various types of glioma cells. In addition, a MACC1-specific shRNA was designed and synthesized in order to investigate the effects of MACC1 inhibition on malignant glioma U251 cells. Following the stable transfection of the MACC1 shRNA into U251 cells, RT-qPCR and western blotting revealed that the MACC1 shRNA could effectively inhibit the expression of MACC1 in shRNA(MACC1) cells. As a consequence of the MACC1 knockdown, U251 cell proliferation, migration and invasion were markedly inhibited, whereas cellular apoptosis was markedly increased. The effects of MACC1 shRNA on cell proliferation inhibition were associated with a downregulation of cyclin D1 and cyclin E; in addition, the observed increase in apoptosis was controlled by the upregulation of cleaved caspase-3 and Bax expression, and the downregulation of Bcl-2 expression. Cell invasion and migration was shown to be suppressed and regulated by the inhibition of MMP-2/-9 activity and expression. These results suggested that inhibition of MACC1 may suppress the growth and metastatic potential of malignant glioma cells, which in turn suggests that MACC1 may be involved in the proliferation, cell cycle arrest, apoptosis, invasion and migration of malignant glioma cells.

Cyclins are positive regulators of cell cycle progression in the cell cycle pathway (22); notably, cyclin E and cyclin D1 are the primary regulators of late G_1_ phase, and contribute to G_1_ phase progression (23) and chromosomal instability (24). It is likely that the cyclins are also involved in tumor initiation and proliferation. A recent study reported that both cyclin E and cyclin D_1_ may have a key role in promoting the growth of glioma cells, as well as their transformation into malignant cells (25). In addition, Zhang *et al* (10) demonstrated that MACC1 shRNA induced G_0_/G_1_ phase cell cycle arrest through cyclin D1 in OVCAR-3 ovarian carcinoma cells. Notably, in the present study, the expression levels of cyclin D_1_ and cyclin E were shown to be downregulated following inhibition of MACC1. Furthermore, the expression levels of cyclin B, which regulates the cell-cycle progression of G_2_/M phase (26), were also downregulated in the shRNA(MACC1) cells. Cell cycle assays using FACS demonstrated that MACC1 shRNA induced U251 cell cycle arrest at G_1_ phase, but not at G_2_/M phase. These results account for the fact that the main regulatory targets of MACC1 in U251 cells were cyclin D_1_ and cyclin E, rather than cyclin B. MACC1 may have either a direct or indirect effect on cyclin D_1_ and cyclin E in order to arrest the cell cycle at the G_1_ phase. Although cyclin B was downregulated by MACC1 shRNA, this alone is not sufficient to arrest the cell cycle at G_2_/M phase.

Apoptosis is mediated through a caspase cascade (27), and the ability of cells to undergo apoptosis is determined by the interaction between pro-survival proteins, such as Bcl-2, and pro-apoptotic proteins, such as Bax (28). Numerous studies have also demonstrated that Bcl-2 overexpression is associated with resistance to conventional radiation and chemotherapeutic agents in the majority of tumor cells, including malignant glioma cells (29–31). This is achieved as Bcl-2 binds to Bax, inhibiting Bax activation (32). In the present study, the expression levels of both cleaved caspase-3 and Bax were shown to be upregulated following MACC1 silencing in U251 cells. Conversely, the expression levels of Bcl-2 were downregulated; thus suggesting that MACC1 shRNA targets Bcl-2 in order to suppress the inhibition of Bax by promoting the release of pro-apoptotic Bax from a Bax/Bcl-2 heterodimeric complex. The released Bax promotes the formation of outer-mitochondrial membrane spanning pores, which triggers the activation of the caspase cascade orchestrated by caspase-3, ultimately inducing apoptosis (33). However, the precise mechanisms by which the knockdown of MACC1 causes cancer cell cycle arrest and apoptosis requires further elucidation.

In addition to controlling the important events of cell proliferation and apoptosis, MACC1 is also involved in tumor cell invasion and metastasis (34). Increased MMP activity has been detected in virtually every type of human cancer, and correlates with advanced stage, invasive and metastatic properties, and poor general prognosis (35). In the present study, the properties of invasion and metastasis were examined using wound healing and transwell assays in the shRNA(MACC1) and shRNA(-) U251 cells. The results demonstrated that the capacity for migration and invasion were markedly decreased in the shRNA(MACC1) cells. In addition, western blotting revealed that the expression levels of MMP-2 and MMP-9 were downregulated. Consistent with these findings, the enzymatic activities of MMP-2 and MMP-9 were inhibited in the shRNA(MACC1) group, as determined by the gelatin zymography assay. The results of the present study suggested that MACC1 is involved in MMP-2/-9-mediated invasion and metastasis. The inhibition of MACC1 expression suppressed the activity of MMP-2 as well as MMP-9, leading to a decreased capacity for invasion and metastasis in malignant glioma cells (35). Furthermore, numerous studies have suggested that MACC1 induces tumor metastasis through the regulation of the hepatocyte growth factor (HGF)/MET signaling pathway (12,36,37). In addition, the MET gene encoding the HGF receptor is a transcriptional target of MACC1 (19). It therefore appears plausible that the downregulation of MMP-2 and MMP-9 by MACC1 shRNA may be also be mediated via the HGF/MET signaling pathway. However, this assumption requires further investigation.

In conclusion, the results of the present study demonstrated that human glioblastomas exhibit increased expression of MACC1. The silencing of MACC1 by shRNA significantly inhibited proliferation, migration and invasion, and promoted apoptosis in U251 human glioma cells. Thus suggesting that MACC1 is a key regulator of these upstream signaling pathways, and may be a potential therapeutic target for malignant gliomas.
